# Distinctive patterns of peripheral neuropathy across the spectrum of plasma cell disorders

**DOI:** 10.1038/s41598-019-53289-w

**Published:** 2019-11-14

**Authors:** Ja Min Byun, Young Nam Kwon, Youngil Koh, Sung-Soo Yoon, Jung-Joon Sung, Inho Kim

**Affiliations:** 1 Department of Internal Medicine, Seoul National University College of Medicine, Seoul National University Hospital, Seoul, South Korea; 2 Department of Neurology, Seoul National University College of Medicine, Seoul National University Hospital, Seoul, South Korea

**Keywords:** Myeloma, Neuropathic pain

## Abstract

Many patients with plasma cell disorders suffer from peripheral neuropathy, but differential diagnosis with chronic inflammatory demyelinating polyneuropathy (CIDP) is difficult. We aimed to (1) identify factors useful for differential diagnosis between peripheral neuropathy associated with plasma cell disorders versus CIDP and (2) determine whether neuropathy presentations and severity varied across the spectrum of different plasma cell disorders. A retrospective chart review of 18 monoclonal gammopathy of unknown significance (MGUS) patients, 15 POEMS syndrome patients and 34 CIDP patients between January 2005 and December 2016 was conducted. The peripheral neuropathy associated with plasma cell disorders seemed to be more sensory oriented compared to CIDP. MGUS patients were significantly older than CIDP patients (median age 70 vs. 59, respectively, *p* = 0.027). POEMS syndrome patients showed significantly higher platelet count at the time of neuropathy presentation compared to CIDP (*p* = 0.028). Lambda type MGUS patients were associated with less severe symptoms compared to POEMS syndrome patients despite harboring lambda monoclonal gammopathy as a common denominator. Kappa type MGUS patients showed predominantly axonal type neuropathy compared to its counterpart and POEMS syndrome. Careful inspection of clinical profiles and symptoms of patients presenting with neuropathy can help to discriminate those with underlying plasma cell disorders. The phenotype of neuropathy, platelet count and age at presentation seem to be the most useful indicators.

## Introduction

Plasma cell disorders are a heterogeneous group of clonal disease characterized by varying amount of plasma cells in the bone marrow and the presence of a monoclonal (M) protein^1^. Plasma cell disorders ranges from subclinical monoclonal gammopathy of unknown significance (MGUS) to malignant systemic disorders such as multiple myeloma and amyloidosis, and POEMS syndrome (polyneuropathy, organomegaly, endocrinopathy, monoclonal gammopathy and skin changes). Interestingly many of the patients with monoclonal gammopathy also suffer from peripheral neuropathy, and the causal association between the two has been previously explored^2,3^. It appears that 85% to 100% of the patients with POEMS syndrome or osteosclerotic variant of multiple myeloma have neuropathy at disease diagnosis^4,5^, while approximately a third of the patients with MGUS suffer from neuropathy^6–8^. Since MGUS, the most common of the plasma cell disorders, is present in over 3–4% of the general population over the age of 50^9^, it is not uncommon to encounter patients with peripheral neuropathy in whom further evaluation reveals a presence of M protein. Other studies have shown that among patients with peripheral neuropathy, especially in patients referred to tertiary hospitals for no other apparent causes, 3–5% showed detectable M protein of varying s^5,10^.

When patients with neuropathy present with overt symptoms of plasma cell disorders, for exampleheart failures or significant weight loss, suspecting an underlying hematologic malignancy is not so difficult. Unfortunately, neuropathy is the sole initial symptoms for many patients, thus underdiagnosis (i.e not testing for the presence of monoclonal gammopathy) or confusion with chronic inflammatory demyelinating polyneuropathy (CIDP) often occur, subsequently leading to inappropriate management. To this end, we selected MGUS patients with neuropathy, POEMS syndrome patients and CIDP patients and compared their clinical characteristics and course to (1) identify factors useful for differential diagnosis between peripheral neuropathy associated with plasma cell disorders versus CIDP and (2) determine whether neuropathy presentations and severity varied across the spectrum of different plasma cell disorders.

## Materials and Methods

This study was carried out at Seoul National University Hospital, which is a tertiary academic center. During the period between January 2005 and December 2016, patients ≥18 years of age with (1) newly diagnosed MGUS with documented neuropathy, (2) newly diagnosed POEMS syndrome and (3) newly diagnosed CIDP without evidence of monoclonal gammopathy (*i*.*e*. negative on serum protein electrophoresis) were identified. The diagnosis of MGUS and POEMS syndrome was made according to the International Myeloma Working Group Criteria (IMWG)^1,11^. CIDP was diagnosed based on the clinical presentation, as judged by a neurologist, and the presence of demyelination on nerve conduction studies (NCS) according to the European Federation of Neurological Societies and Peripheral Nerve Society (EFNS/PNS)^12,13^. Those with previous history of cancer or autoimmune disease requiring treatment with immunomodulatory agents were excluded from analyses. Patients with history of diabetes were also excluded for possibility of confounding. In the end, a total of 18 MGUS patients, 15 POEMS syndrome patients and 34 CIDP patients, with complete set of data including clinical physical examinations, electrophysiologic studies, and laboratory test results, were enrolled. NCS was performed using Viking electromyography machine (Neuroscreen, Viking Select and Sierra Wave machines). For motor, median, ulnar, peroneal and tibial NCS was performed to measure terminal latency, nerve conduction velocity (NCV), compound muscle action potential (CMAP) amplitude, and F wave latency. For sensory, median, ulnar and sural NCS was performed using antidromic methods, and sensory nerve action potential (SNAP) amplitude and NCV were measured. The differences between groups were assessed using a Student’s t-test or one-way analysis of variance for continuous variables, and Pearson chi-square test for categorical variables, as indicated. The overall survival (OS) was defined as the time from monoclonal gammopathy or CIDP diagnosis to death from any cause. If patients survived, OS was censored on the last date of follow-up. All data were analyzed using the Statistical Package for the Social Sciences software (IBM^®^ SPSS^®^Statistics, version 22.0). *p* values of <0.05 were considered statistically significant. The study was conducted in compliance with all national and international ethical standards for research with humans and for research using radiopharmaceuticals. This study was conducted according to the Declaration of Helsinki and was approved by the institutional review board of Seoul National University Hospital (IRB No. H-1605-152-768) and patients gave written informed consent before being enrolled. All authors had access to the study data and reviewed and approved this study.

## Results

### Clinical characteristics of the enrolled patients

**Figure 1:**
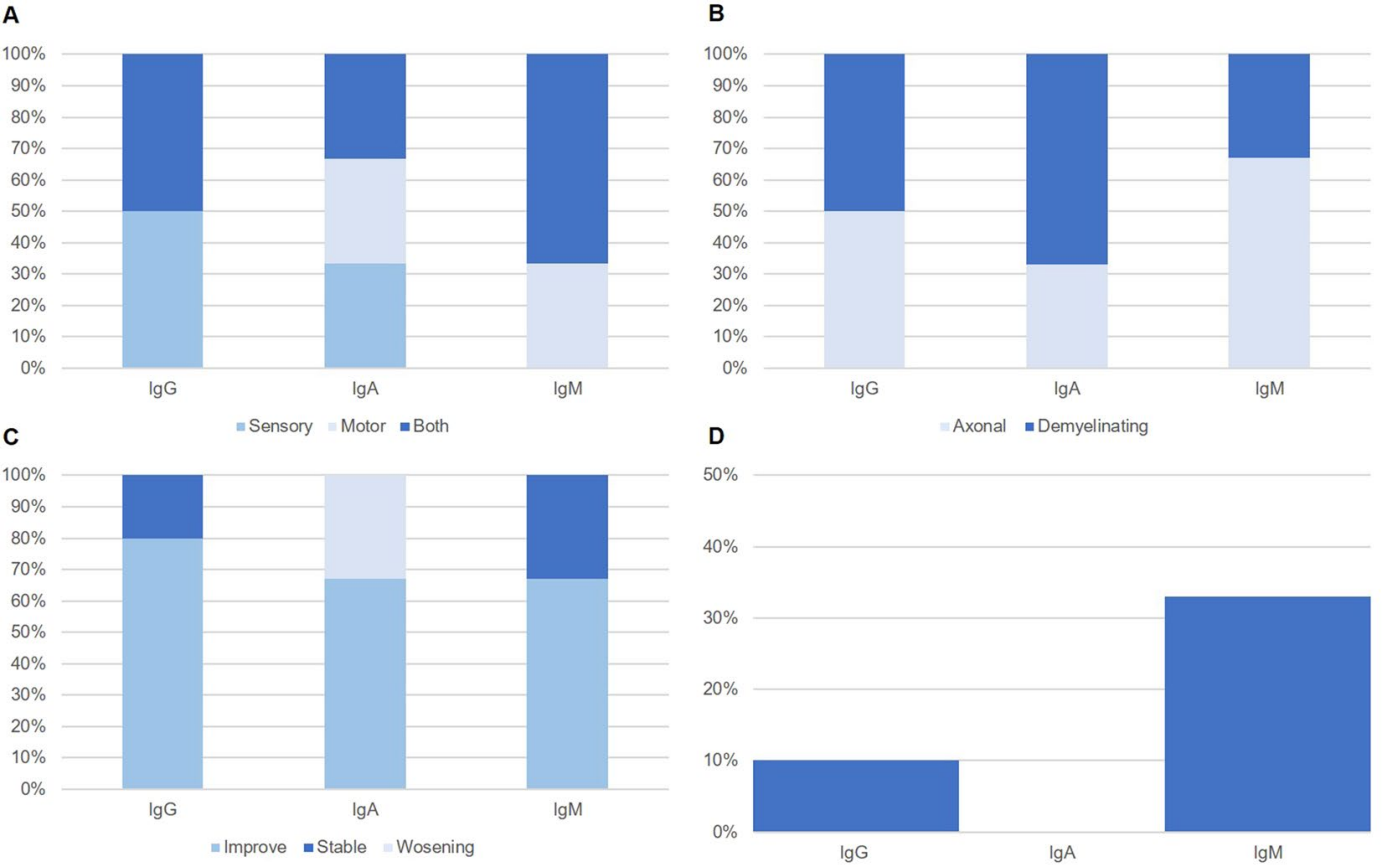
Clinical findings in the 3 main peak groups of MGUS. (**A**) Clinical presentation. (**B**) Nerve conduction studies finding. (**C**) Symptom evolution. (**D**) Percentage of plasma cell neoplasm development. MGUS, monoclonal gammopathy of unknown significance.

The baseline characteristics of all enrolled patients are presented in Table 1. In all groups, males were predominant. During the median follow-up of 49 months, 2 MGUS patients developed overt hematologic malignancies: 1 case of Waldenstrom macroglobulinemia and 1 case of AL amyloidosis (Table 2 and Fig. 1). Both of them showed malignant transformation 1 year of neuropathy development, and were associated with worsening neuropathic symptom at the diagnosis of hematologic malignancy. The treatment and response to treatment, along with survival data are shown in Table 2. Disease related death occurred in only 1 patient among the whole population.

**Table 1:** Clinical characteristics of all enrolled patients.

Characteristics		MGUS			POEMS	CIDP	p*
(N, %)		lambda	kappa	total			
N		6	12	18	15	34	NA
Age at diagnosis	Median (years, range)	71 (52–76)	71 (53–81)	70 (52–81)	56 (31–67)	59 (21–83)	0.007
Sex	Male	4 (66.7)	9 (75.0)	13 (72.2)	8 (53.3)	24 (70.6)	0.429
Ig type	IgG	3 (50.0)	7 (58.3)	10 (55.6)	4 (26.7)	NA	NA
	IgA	2 (33.3)	1 (8.3)	3 (16.7)	6 (40.0)	NA	
	IgM	0	3 (25.0)	3 (16.7)	0	NA	
	Others	1 (16.7)	1 (8.3)	2 (11.1)	5 (33.3)	NA	
Light chain	Kappa	0	12 (100)	6 (33.3)	0	NA	NA
	Lambda	6 (100)	0	12 (66.7)	15 (100)	NA	
Laboratory results	Serum M protein (g/dL)	0.3 (0.4)	0.6 (0.5)	0.5 (0.5)	0.3 (0.4)	0	NA
(mean ± SD)	beta2-MG (mg/L)	2.0 (0.5)	2.5 (1.1)	2.4 (0.9)	4.4 (3.5)	NA	NA
	Hemoglobin (g/dL)	13.6 (2.5)	12.5 (1.9)	12.9 (2.2)	12.5 (2.4)	13.3 (2.0)	0.602
	Platelets (10^3^/L)	204 (77)	246 (65)	231 (70)	341 (157)	252 (81)	0.011
	Calcium (mg/dL)	8.6 (0.6)	9.1 (0.6)	8.9 (0.6)	7.8 (0.8)	9.2 (0.5)	<0.001
	Creatinine (mg/dL)	0.9 (0.2)	0.8 (0.2)	0.8 (0.2)	1.0 (0.5)	1.3 (1.9)	0.556
	Albumin (mg/dL)	3.7 (0.7)	3.7 (0.7)	3.7 (0.7)	3.3 (0.6)	4.0 (0.4)	<0.001
	LDH (IU/L)	555.5 (591.8)	236.3 (110.0)	342.7 (323.4)	118.1 (44.7)	293.6 (179.0)	0.163
	BM plasma cell (%)	1.3 (1.4)	3.1 (2.8)	2.6 (2.5)	3.3 (2.3)	NA	NA

*p value for MGUS vs POEMS vs CIDP.

MGUS, monoclonal gammopathy of unknown significance; POEMS, Polyneuropathy, organomegaly, endocrinopathy, monoclonal gammopathy, and skin changes syndrome; CIDP, chronic inflammatory demyelinating polyneuropathy; SD, standard deviation; MG, microglobulin; LDH, lactate dehydrogenase; BM, bone marrow; NA, not applicable.

**Table 2:** Treatment and clinical course.

Parameter	MGUS			POEMS	CIDP
(N, %)	lambda	kappa	total		
**Treatment**	6	12	18	15	34
Observation/symptomatic care	3 (50.0)	6 (50.0)	9 (50.0)	1 (6.7)	9 (26.5)
Chemotherapy	0	1 (8.3)	1 (5.6)	14 (93.3)	0
Immunomodulation*	3 (50.0)	5 (41.7)	8 (44.4)	1 (6.7)**	25 (73.5)
**Neuropathy response to treatment**					
Improved	1 (16.7)	5 (41.7)	6 (33.3)	4 (26.7)	17 (50.0)
Stable	4 (66.7)	6 (50.0)	10 (55.6)	6 (40.0)	11 (32.4)
Worsening	1 (16.7)	1 (8.3)	2 (11.1)	5 (33.3)	6 (17.6)
**Plasma cell neoplasm development**					
*Number*	1 (16.7)	1 (8.3)	2	0	0
*Presenting symptom*					
Renal function deterioration	0	1	NA	NA	NA
Anemia development	0	1			
Worsening of neuropathy	1	1			
*Time to neoplasm development* (*months*)	8	6			
**Overall survival**					
Median (months)	NR	NR	NR	NR	110
Mean (±SD), months	68 (40)	42 (32)	51 (36)	59 (45)	61 (32)
Total death/disease related death	0/0	2 (16.7)/0	2 (11.1)/0	5 (33.3)/1 (6.7)	4 (11.8)/0

*Immunomodulation refers to use of intravenous immunoglobulin, rituximab, azathioprine, mycophenolate mofetil or cyclophosphamide. The use of oral prednisone was not included. Some of the MGUS and POEMS syndrome patients were initially misdiagnosed as CIDP, and thus were treated with immunomodulation.

**One POEMS syndrome patient was initially diagnosed as CIDP and was subjected to intravenous immunoglobulin. Lack of response led to re-evaluation and the patient was subsequently diagnosed as POEMS syndrome and received chemotherapy. Thus the percentage for this column exceeds 100%.

MGUS, monoclonal gammopathy of unknown significance; POEMS, Polyneuropathy, organomegaly, endocrinopathy, monoclonal gammopathy, and skin changes syndrome; CIDP, chronic inflammatory demyelinating polyneuropathy; NA, not applicable; NR, not reached.

### Comparing MGUS with CIDP

Overall MGUS patients tended to be older than CIDP patients (*p* = 0.027, Table 1). Also, MGUS patients had more pure sensory symptoms (44.4% of all MGUS patients vs. 8.8% of CIDP patients, *p* = 0.003) compared to CIDP. When different subtypes of MGUS was considered, both lambda type MGUS (*p* = 0.009) and kappa type MGUS (*p* = 0.010) had predominantly sensory type neuropathy compared to CIDP (Table 3). Interestingly, when compared to lambda type MGUS, CIDP patients seemed to show more muscle atrophy (0% in lambda type MGUS vs. 41.2% in CIDP, *p* = 0.051).

**Table 3:** Neurologic manifestations.

Parameter	MGUS			POEMS	CIDP	p^*^
(N, %)	lambda	kappa	total			
**Electrophysiological findings**						
***NCS pattern***						
Axonal	0	9 (75.0)	9 (50.0)	3 (20.0)	0	<0.001
Demyelinating	6 (100)	3 (25.0)	9 (50.0)	12 (80.0)	34 (100.0)	
***Clinical phenotype***						
Pure sensory	3 (50.0)	5 (41.7)	8 (44.4)	2 (13.3)	3 (8.8)	0.003
Pure motor	0	1 (8.3)	1 (5.6)	0	1 (2.9)	0.646
Sensorimotor	3 (50.0)	6 (50.0)	9 (50.0)	13 (86.7)	30 (88.2)	0.004
Symmetric involvement	4 (66.7)	7 (58.3)	11 (61.1)	11 (73.3)	27 (79.4)	0.367
**Neurologic symptoms**						
***Sensory***						
Dysthesia	5 (83.3)	11 (91.7)	16 (88.9)	14 (93.3)	32 (94.1)	0.785
Hypesthesia to temperate & pinprick	2 (33.3)	5 (41.7)	7 (38.9)	6 (40.0)	18 (52.9)	0.538
Hypesthesia to vibration & position	3 (50.0)	5 (41.7)	8 (44.4)	8 (53.3)	22 (64.7)	0.357
Pain, allodynia	2 (33.3)	2 (16.7)	4 (22.2)	7 (46.7)	6 (17.6)	0.093
***Motor***						
Gait disturbance	2 (33.3)	9 (75.0)	11 (61.1)	6 (40.0)	21 (61.8)	0.333
Ataxia	2 (33.3)	9 (75.0)	11 (61.1)	4 (26.7)	21 (61.8)	0.058
***Physical examinations***						
Muscle atrophy	0	4 (33.3)	4 (22.2)	4 (26.7)	14 (41.2)	0.325
MRC sum^*^	57.7 (4.1)	54.5 (8.1)	55.6 (7.0)	51.7 (6.5)	54.4 (5.3)	0.178
Initial mRS^*^	1.8 (1.0)	2.8 (1.4)	2.4 (1.3)	3.0 (1.3)	2.6 (1.2)	0.431

*p value for MGUS vs POEMS vs CIDP.

MGUS, monoclonal gammopathy of unknown significance; POEMS, Polyneuropathy, organomegaly, endocrinopathy, monoclonal gammopathy, and skin changes syndrome; CIDP, chronic inflammatory demyelinating polyneuropathy; NCS, nerve conduction study; MRC, Medical Research Council; mRS, modified Rankin scale.

Eight out of 18 MGUS patients were initially diagnosed as CIDP and were treated as such. These 8 patients who were initially misdiagnosed showed more symmetric manifestation of neuropathy (30% vs. 100%, respectively, *P* = 0.002), more gait disturbance (40% vs. 87.5%, *p* = 0.040), and higher B2MG level at diagnosis (mean 2.1 mg/L vs 4.2 mg/L, *p* = 0.014) compared to rest of the MGUS patients.

### Comparing POEMS syndrome with CIDP

Next, we compared CIDP patients with POEMS syndrome patients. As the very diagnosis suggests, all of the CIDP patients showed demyelinating polyneuropathy on NCS while 3 of the POEMS syndrome patients showed axonal polyneuropathy (*p* = 0.025; Table 3). POEMS syndrome patients more often complained of allodynia (46.7% of POEMS syndrome vs. 17.6% of CIDP patients, *p* = 0.034), while CIDP patients more often suffered from ataxia (26.7% of POEMS syndrome vs. 61.8% of CIDP patients, *p* = 0.024) POEMS syndrome patients were associated with higher platelet count (*p* = 0.028), and lower serum protein level (*p* = 0.009) and LDH (*p* = 0.030) compared to CIDP patients at the time of diagnosis.

One of the POEMS syndrome patient was initially misdiagnosed as having CIDP. This patient was initially treated with steroids and immunoglobulin but showed worsening symptoms and was later re-diagnosed as POEMS syndrome.

### Comparing lambda type MGUS with POEMS syndrome

Since POEMS syndrome is exclusively associated with lambda type M protein, we first compared POEMS syndrome patients with lambda type MGUS. The 6 lambda type MGUS did not meet the IMWG criteria for the diagnosis of POEMS syndrome for none of them met the major criteria (i.e. osteosclerotic or mixed sclerotic/lytic lesion on plain films or computed tomography, or, Castleman disease, or, elevated serum or plasma vascular endothelial growth factor levels). Compared to POEMS syndrome patients, lambda type MGUS patients tended to be older (*p* = 0.021; Table 1). The lambda type MGUS patients manifested predominantly sensory dominant neuropathy (*p* = 0.022; Table 3), but generally less severe grade of neuropathy evidence by lower initial modified Rankin scale (MRS) (mean score ± standard deviation, 1.8 ± 1.0 for MGUS vs. 3.0 ± 1.3 for POEMS syndrome, *p* = 0.046) and higher Medical Research Council (MRC) sum score (mean score ± standard deviation, 57.7 ± 4.1 for MGUS vs. 51.7 ± 6.5 for POEMS syndrome, *p* = 0.023). In both groups, demyelinating type polyneuropathy was more common. The lambda type MGUS patients were associated with lower platelet count at diagnosis (*p* = 0.021), and higher serum calcium (*p* = 0.023) and LDH (*p* = 0.048) compared to POEMS syndrome patients.

### Comparing kappa type MGUS with POEMS syndrome

Compared to POEMS syndrome patients, kappa type MGUS patients also tended to be older (*p* = 0.001; Table 1). Moreover, the kappa type MGUS also manifested pure sensory-oriented neuropathy compared to POEMS syndrome (*p* = 0.030; Table 3). Interestingly, kappa type MGUS patients were more often associated with axonal polyneuropathy compared to POEMS syndrome patients (*p* = 0.004). More patients with kappa type MGUS suffered from ataxia (*p* = 0.013) and accordingly more gait disturbance compared to POEMS syndrome patients, though the difference failed to reach statistical significance (*p* = 0.069). The kappa type MGUS patients showed lower platelet count at diagnosis (*p* = 0.048), and higher serum calcium (*p* < 0.001) and LDH (*p* = 0.030).

As shown in Table 4, when the specifics of NCS are considered the difference between these two entities become more prominent. When motor NCS is considered, kappa type MGUS patients had significantly faster NCVs (median, *p* = 0.004; tibial, *p* = 0.013), higher CMAP amplitude (tibial, *p* < 0.001) and shorter F wave latencies (median *p* = 0.036; tibial, *p* = 0.002) at median and/or tibial nerves compared to their POEMS syndrome counterparts. Furthermore, the NCVs were faster (median, p = 0.001; sural, p = 0.001) and SNAP amplitude was lower (median, *p* < 0.001) at median and/or sural sensory nerves in patients with kappa type MGUS compared to patients with POEMS syndrome.

**Table 4:** Nerve conduction data.

	MGUS		POEMS	CIDP	*p*
	lambda	kappa (A)	(B)		(for A vs B)
**Motor conduction study**	6	12	15	34	
***Median nerve***					
Terminal latency index	4.850 (1.601)	5.067 (2.559)	4.500 (1.292)	5.183 (1.956)	0.699
NCV (m/s)	39.083 (10.171)	47.067 (12.447)	33.267 (9.565)	43.536 (10.574)	0.004
CMAP amplitude (mV)	7.183 (2.967)	8.131 (3.798)	5.439 (4.515)	7.355 (4.298)	0.271
F wave latency (ms)	33.933 (5.543)	29.907 (4.874)	40.611 (8.354)	35.237 (9.712)	0.036
***Tibial nerve***					
Terminal latency index	7.000 (3.335)	5.481 (2.687)	5.522 (0.578)	6.419 (2.115)	0.219
NCV (m/s)	32.700 (8.028)	40.000 (8.234)	30.667 (4.664)	34.895 (8.3188)	0.013
CMAP amplitude (mV)	2.308 (1.580)	6.878 (5.437)	0.840 (2.013)	4.103 (4.014)	<0.001
F wave latency (ms)	60.400 (7.150)	49.100 (4.798)	63.720 (7.629)	57.358 (9.810)	0.002
**Sensory conduction study**					
***Median nerve***					
NCV (m/s)	45.667 (3.786)	50.467 (3.091)	40.333 (4.950)	47.300 (6.739)	0.001
SNAP amplitude (uV)	11.683 (13.882)	40.581 (31.436)	9.656 (12.558)	15.867 (15.510)	<0.001
No response (n)	3	1	9	16	NA
***Sural nerve***					
NCV (m/s)	34.375 (2.326)	37.875 (4.225)	30.714 (4.250)	34.233 (5.001)	0.001
SNAP amplitude (uV)	5.658 (5.191)	10.457 (12.131)	4.667 (8.085)	5.955 (8.193)	0.103
No response (n)	5	2	9	16	NA

Data are expressed as mean (**±**standard deviation).

MGUS, monoclonal gammopathy of unknown significance; POEMS, Polyneuropathy, organomegaly, endocrinopathy, monoclonal gammopathy, and skin changes syndrome; CIDP, chronic inflammatory demyelinating polyneuropathy; NCV, nerve conduction velocity; CMAP, compound muscle action potential; SNAP, sensory nerve action potential; NA, not applicable.

## Discussion

Peripheral neuropathy is a well-recognized complication across the spectrum of different plasma cell disorders, but represents a challenging clinical problem in terms of diagnosis and treatment. In patients with other additional symptoms pointing to the diagnosis of plasma cell disorders with known causal relationship with neuropathy, namely POEMS syndrome, clinical suspicion may not be so difficult. However, in majority of the cases peripheral neuropathy is the sole complaint and it is not easy to immediately suspect an underlying monoclonal gammopathy in everyday practice. Furthermore, even when the presence of M protein is confirmed, distinguishing patients in whom the M protein is the cause of peripheral neuropathy from patients in whom the presence of M protein is an incidental finding and unrelated to the neuropathy poses an another challenge. To this end, we have carried out this study to first compare manifestations and clinical courses of peripheral neuropathy with underlying plasma cell disorder versus pure CIDP patients, then to compare patients with different plasma cell disorders with peripheral neuropathy, and made some interesting discoveries.

In general, peripheral neuropathy related to plasma cell disorders seem to be more sensory oriented. More specifically, POEMS syndrome patients were significantly more associated with allodynia compared to CIDP patients. This agrees with previous study reporting POEMS syndrome patients more frequently complained of being in pain compared to CIDP patients^14^. Also, MGUS patients were much older than others. The median age at diagnosis for MGUS with peripheral neuropathy patients was 70, suggesting underlying plasma cell disorder should be ruled out for older patients with unexplained polyneuropathy. The difference in median age at diagnosis could also explain the difference in overall survival. Since disease related mortality was documented in only 1 patient, and was associated with treatment related mortality during autologous stem cell transplantation for POEMS syndrome, it can be safely assumed that the survival curves represent natural deaths. Meanwhile, POEMS syndrome patients had higher platelet count at the time of diagnosis. This finding coincides with previous report by Naddaf *et al*.^15^, who reported that 53.7% of POEMS patients showed thrombocytosis with median platelet count of 467 × 10^3^/L while only 1.5% of CIDP patients showed thrombocytosis with median platelet count of 275 × 10^3^/L. Likewise, the mean platelet count for POEMS syndrome patients was 341 × 10^3^/L compared to 252 × 10^3^/L for CIDP patients (*p* = 0.028). The one patient who was initially mistaken as CIDP also had a high platelet count (719 × 10^3^/L). All in all, it seems that platelet count can be a helpful indicator to prompt physicians to consider POEMS syndrome in patients who are thought to have CIDP.

Among the patients with MGUS, 8 patients were initially misdiagnosed as CIDP (Table 5). In these patients, the presence of monoclonal gammopathy was checked only after they failed to respond to steroids, intravenous immunoglobulins (IVIG) and immunomodulating agents, namely azathioprine (n = 4) and rituximab (n = 4). The fact that these patients underwent multiple lines of rather costly yet ineffective treatment, along with the fact that the median interval time between initial CIDP diagnosis and MGUS diagnosis was 19 months (6–49 months), highlights that clinical suspicion is the key to timely diagnosis. Nevertheless, in some cases, distinguishing MGUS from CIDP would be not easy using clinical manifestations only. Therefore, checking serum paraproteins is warranted for patients suspected with CIDP.

**Table 5:** Clinical characteristics of the 8 MGUS patients initially misdiagnosed as having CIDP.

Patient	Treatment for CIDP	Peripheral neuropathy			Interval between CIDP to MGUS diagnosis	Monoclonal gammopathy	
		Symmetry	Presentation	Type		Heavy chain	Light chain
1	Steroids, IVIG, azathioprine, rituximab	Yes	Sensorimotor	Demyelinating	49 months	IgA	Lambda
2	Steroids, IVIG, azathioprine	Yes	Pure sensory	Demyelinating	38 months	IgG	Lambda
3	Steroids, IVIG, azathioprine	Yes	Sensorimotor	Demyelinating	6 months	LCD	Lambda
4	Steroids, IVIG, rituximab	Yes	Sensorimotor	Demyelinating	25 months	IgM	Kappa
5	Steroids, IVIG, MMF, rituximab	Yes	Sensorimotor	Axonal	37 months	IgM	Kappa
6	Steroids, IVIG, rituximab	Yes	Sensorimotor	Demyelinating	6 months	IgG	Kappa
7	Steroids, IVIG, azathioprine	Yes	Pure sensory	Axonal	14 months	LCD	Kappa
8	Steroids, IVIG, azathioprine	Yes	Sensorimotor	Demyelinating	6 months	IgA	Kappa

MGUS, monoclonal gammopathy of unknown significance; CIDP, chronic inflammatory demyelinating polyneuropathy; IVIG, intravenous immunoglobulin; MMF, myphenolate mofetil; LCD, light chain disease.

Further touching on the subject of MGUS with peripheral neuropathy, only 16.7% of our patients were of IgM heavy chain subtype. Traditionally, it is believed that the type of M protein in monoclonal gammopathy associated peripheral neuropathy is mostly IgM, while IgG, or IgA neuropathies are less common^2,16,17^. In one report, IgM constituted 60% of the neuropathies associated with monoclonal gammopathy followed by IgG (30%), and IgA (10%)^17^. Generally, it is thought that IgM monoclonal gammopathy is associated with peripheral neuropathy presenting as distal acquired, demyelinating, symmetric neuropathy with M protein (DADS-M)^8,18^, while non-IgM monoclonal gammopathy patients show a more diverse phenotypes including the length-dependent sensorimotor axonal peripheral neuropathy^19^. In addition, IgM MGUS was known to be associated with anti-myelin-associated glycoprotein (MAG) neuropathy^20^. However, due to the small population of IgM MGUS patients in our cohort, we could not assess the association of anti-MAG antibody in this study. Since previous studies are heavily oriented towards Caucasian populations, we cannot say for certain if such discrepancy between our and historical data originated from ethnical difference or due to the sample size of our cohort. This finding should be further validated from a larger cohort of East Asian patients.

Compared to heavy chains, the role of light chains in the pathophysiology of monoclonal gammopathy associated with peripheral neuropathy has been less explored. In this context, we were curious if lambda type MGUS with neuropathy would be similar to POEMS syndrome, which harbors an exclusively lambda type monoclonal gammopathy. Both plasma cell disorders were more frequently associated with demyelinating type peripheral neuropathy. However despite sharing lambda light chain as a common denominator, POEMS syndrome patients seemed to have more debilitating disease, evident by higher MRS score and lower MRC sum score. Meanwhile, MGUS patients showed higher serum calcium and LDH levels at the time of diagnosis. On the other hand, kappa type monoclonal gammopathy showed some distinct manifestations compared to its lambda counterparts and POEMS syndrome. Namely, these patients showed predominantly axonal neuropathy or mild demyelinating neuropathy which does not satisfy the definition of demyelinating neuropathy. Also as shown in Table 4, kappa type MGUS patients suffered from similar severity of symptoms compared to POEMS syndrome patients. Considering the fact that there were no differences in the composition of heavy chains between the groups, light chain may be the culprit for such difference.

In conclusion, careful inspection of clinical profiles and symptoms of patients presenting with neuropathy might be helpful to discriminate those with underlying plasma cell disorders. The pattern of neuropathy, platelet count and age at presentation could be useful indicators. Although our study is limited by the retrospective nature and small number of patients included, we provide important findings that can be readily implanted in everyday practice.
